# Comparative Evaluation of 0.2 percent Chlorhexidine and Magnetized Water as a Mouth Rinse on *Streptococcus mutans* in Children

**DOI:** 10.5005/jp-journals-10005-1108

**Published:** 2011-04-15

**Authors:** Nidhi Gupta, Manohar Bhat

**Affiliations:** 1 Senior Lecturer, Department of Pedodontics and Preventive Dentistry, Santosh Dental College, Ghaziabad, Uttar Pradesh, India; 2 Professor and Head, Department of Pedodontics and Preventive Dentistry, Jaipur Dental College, Jaipur, Rajasthan, India

**Keywords:** S. *mutans*, Magnetized water, Chlorhexidine mouthrinse (0.2%).

## Abstract

Chlorhexidine (0.2%) is a ‘Gold Standard’ which is commercially available and effective mouth rinse which inhibits supragingival plaque formation and also reduction in the population of S. *mutans* after rinsing daily. Lately, it has been proved that the force of magnetism has a great influence on the living organism. Magnetized water has its effect on human body when taken regularly for a considerable period but its use in dentistry is still lacking.

**Aim:**

This study was done to evaluate antibacterial efficacy and effect of dosage, frequency and duration of commercially available 0.2% chlorhexidine mouth rinse and conventionally prepared magnetized water on colony count of S. *mutans.*

**Materials and methods:**

A total of 50 subjects were selected between the age group of 5 to 12 years. A baseline sample was collected before starting with rinses. Then the subjects were divided in four major groups. Group I was chlorhexidine, group II was subdivided into group IIA and group IIB which were magnetized water groups (24 hours of magnetization) rinsing for 1 minute and 3 minutes respectively and group III was magnetized water (72 hours of magnetization) group rinsing for 3 minutes. The samples were collected and sent to microbiological laboratory for *S. mutans* count.

**Results:**

The obtained values of all the groups were subjected to statistical analysis.

**Conclusion:**

The reduction of *S. mutans* count of group III was almost in par with group I.

## INTRODUCTION

The removal of supragingival and subgingival bacterial bio-film is a decisive component in the prevention and treatment of dental caries and periodontal diseases.

The microorganisms in bacterial plaque cause inflammatory periodontal disease. For, this reason plaque control plays a significant role in the prevention of caries, gingivitis and periodontitis. Both mechanical procedures and local che-motherapeutics (Cummins 1997)^1^ are used for this purpose.

Chlorhexidine gluconate, a cationic bis-biguanide was introduced for human use in 1957 in Great Britain.

Chlorhexidine (0.2%) mouthrinse has also shown antibacterial efficacy. C Rindom, WW Briner and H Loe (1976)^2^ found a reduction of 30 to 50% in the population of *S. mutans* after rinsing with 10 ml of 0.2% chlorhexidine mouthrinse once daily.

Magnetism is well known in the field of physics. Magnets prove to be strong safeguard against illness and serve as a highly beneficial preventive device. When water passes through the magnetic field, it undergoes certain changes. The magnetic field alters the electrical characteristics of hydrogen ions as well as minerals.

The force of magnetism has a great influence on living organism. When a permanent magnet is kept in continuous contact with water, for considerable time, the water is not only influenced by the magnetic flux of magnet, but also becomes magnetized and acquires magnetic properties. Best results are achieved when water is magnetically treated just prior to use.^3^ Since many researches have been done with the use of magnets in medical field, its use in dentistry is still lacking.

## AIMS AND OBJECTIVES

1. To evaluate and compare antibacterial efficacy of commercially available 0.2% chlorhexidine mouth rinse and conventionally prepared magnetized water on *S. mutans.*
2. To compare and evaluate that dosage, frequency and duration of use of 0.2% chlorhexidine mouth rinse and magnetized water have any effect on colony count of *S. mutans.*

## MATERIALS AND METHODS

This study was conducted in the year 2007-2008 at Arya Orphanage, Pataudi House, Darya Ganj, New Delhi.

### Selection Criteria

Total sample size of 50 children was selected between the age group of 5 to 12 years. The study was conducted over a period of 1 week.

### Subject Selection Criteria

- Systemically healthy patients
- No fixed or removable orthodontic appliances or removable prosthesis
- No history of antibiotic therapy in the subjects within previous 3 months
- No use of chlorhexidine mouth wash or magnetized water as oral rinse earlier
- No history of oral prophylaxis done for atleast 3 months prior to study.

After selection oral prophylaxis of all the subjects was done using ultrasonic scaler. Then the subjects were instructed to abstain from any oral hygiene measures for next 24 hours.

Baseline saliva sample was collected by spitting method in sterile sample collecting bottles for all the subjects.

Subjects were then divided into three major groups (Flow Chart 1).

**Flow Chart 1:**
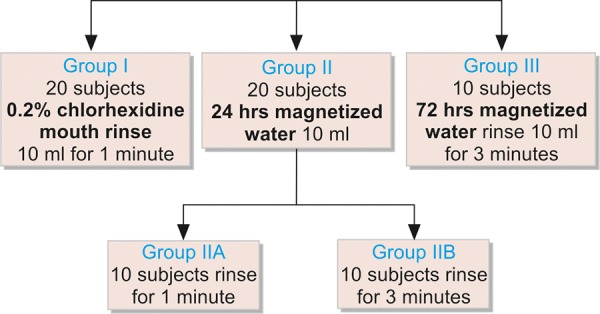
Division of subjects

After collecting baseline samples, the subjects were given the respective mouth rinse as per the groups and were asked to rinse as instructed under supervision and the saliva sample were again collected.

The subjects were then asked to start maintaining there oral hygiene as regular. The same procedure was repeated on day 1 evening.

All the 7 days, the same procedure was followed for all the groups under supervision and the sample collection was done under aseptic conditions.

### Method of Magnetizing Water

RO water was taken in glass bottles and was kept over the magnets for 24 and 72 hours for magnetization.

To check for the magnetization, of the 3 samples (RO water, RO water 24 hours magnetized, RO water 72 hours magnetized) were sent to ‘Metropollis laboratory’ to check for pH and electrical conductivity which reported as follows:

**Table:** 

*Type of water*		*pH*		*Electrical* *conductivity*	
RO water―normal		7.2		25.1	
Magnetized water―24 hours		7.5		24.8	
Magnetized water―72 hours		7.98		11	

### Days of Sample Collection

For Saliva

Day 1―baseline, morning and evening

Day 4―evening

Day 7―morning and evening.

The samples were collected in sterile sample bottles to check for the *S. mutans* count and were carried in the ice box containing ice (as transport media) to microbiology laboratory where the culture plates were inoculated for the *S. mutans* count.

### Mutans Sanguis Agar: Himedia

This agar is recommended for differentiation of *S. mutans* and *S. sanguis* associated with oral microflora.

*S. mutans* forms rough, heaped, irregular colonies resembling frosted glass. Mostly crumbly which are white, gray or yellow in color and 0.5 and 2 mm in diameter.

## RESULTS

Table 1 shows the mean and standard deviation values at various levels of all the groups. Tables 2 to 6 show a student t-test to compare the differences at various levels of all the groups.

## DISCUSSION

The surface of the oral cavity is constantly colonized by microorganisms. One milliliter of whole saliva may contain more than 200 million organism representing more than 250 different species.

*Streptococcus* constitutes an essential part of the mi-croflora which constantly colonize the mucous membrane and the teeth. The *streptococci* in the oral cavity comprise *S. sanguis, S. mitis, S. salivarius, S. intermedius* and other *streptococci* of which *mutans streptococci* especially *S. mutans* and *S. sobrinus* are maximum.

**Table 1:** **Table 1:** Mean and standard deviation values of S. *mutans* (in cfu/ml) at various levels of groups I, IIA, IIB and III

*Groups*		*Levels*											
		*Baseline*		*1st day morning*		*1st day evening*		*4th day evening*		*7th day morning*		*7th day evening*	
Group I		140.25 ± 48.02		2.75 ± 10.86		00.00 ± 00.00		00.00 ± 00.00		00.00 ± 00.00		00.00 ± 00.00	
Group IIA		117.50 ± 37.16		108.50 ± 39.69		103.50 ± 29.75		89.00 ± 24.98		82.50 ± 29.00		70.50 ± 32.59	
Group IIB		165.00 ± 45.00		144.50 ± 48.70		120.50 ± 39.90		88.00 ± 24.82		71.50 ± 16.29		64.50 ± 22.07	
Group III		160.00 ± 43.59		160.00 ± 43.59		97.50 ± 30.52		65.00 ± 16.58		39.00 ± 16.25		30.00 ± 10.00	

**Table 2:** **Table 2:** Statistical comparison (by unpaired t-test) of S. *mutans* (in n × 10^3^ cfu/ml) of mean change at various levels between groups I and IIA

*Groups*		*Levels*									
		*1st day morning*		*1st day evening*		*4th day evening*		*7th day morning*		*7th day evening*	
Group I		137.50 ± 53.70		140.25 ± 49.27		140.25 ± 49.27		140.25 ± 49.27		140.25 ± 49.27	
Group IIA		9.00 ± 16.80		14.00 ± 20.79		28.50 ± 33.25		35.00 ± 37.78		47.00 ± 39.59	
p-value		< 0.001		< 0.001		< 0.001		< 0.001		< 0.001	
Significance		HS		HS		HS		HS		HS	

HS - highly significant

**Table 3:** **Table 3:** Statistical comparison (by unpaired t-test) of *S.mutans* (in cfu/ml) of mean change at various levels between groups I and IIB

*Groups*		*Levels*									
		*1st day morning*		*1st day evening*		*4th day evening*		*7th day morning*		*7th day evening*	
Group I		137.50 ± 53.70		140.25 ± 49.27		140.25 ± 49.27		140.25 ± 49.27		140.25 ± 49.27	
Group IIB		20.50 ± 28.52		44.50 ± 32.18		77.00 ± 36.98		93.50 ± 53.07		100.50 ± 60.07	
p-value		< 0.001		< 0.001		< 0.01		< 0.05		> 0.05	
Significance		HS		HS		S		S		NS	

HS - highly significant, S - significant; NS - not significant

**Table 4:** **Table 4:** Statistical comparison (by unpaired t- test) of S. *mutans* (in cfu/ml) of mean change at various levels between groups I and III

*Groups*		*Levels*									
		*1st day morning*		*1st day evening*		*4th day evening*		*7th day morning*		*7th day evening*	
Group I		137.50 ± 53.70		140.25 ± 49.27		140.25 ± 49.27		140.25 ± 49.27		140.25 ± 49.27	
Group III		0.00 ± 0.00		62.50 ± 37.73		95.00 ± 36.89		121.00 ± 43.38		130.00 ± 42.16	
p-value		< 0.001		< 0.001		< 0.001		< 0.001		> 0.05	
Significance		HS		HS		HS		HS		NS	

HS - highly significant, NS - not significant

**Table 5:** **Table 5:** Statistical comparison (by unpaired t-test) of S. *mutans* (in cfu/ml) of mean change at various levels between groups IIA and IIB

*Groups*		*Levels*									
		*1st day morning*		*1st day evening*		*4th day evening*		*7th day morning*		*7th day evening*	
Group IIA		9.00 ± 16.80		14.00 ± 20.79		28.50 ± 33.25		35.00 ± 37.78		47.00 ± 39.59	
Group IIB		20.50 ± 28.52		44.50 ± 32.18		77.00 ± 36.98		93.50 ± 53.07		100.50 ± 60.07	
p-value		> 0.05		< 0.05		< 0.01		< 0.05		< 0.05	
Significance		NS		S		S		S		S	

NS - not significant, S - significant

**Table 6:** **Table 6:** Statistical comparison (by unpaired t-test) of S. *mutans* (in cfu/ml) of mean change at various levels between groups IIB and III

*Groups*		*Levels*									
		*1st day morning*		*1st day evening*		*4th day evening*		*7th day morning*		*7th day evening*	
Group IIB		20.50 ± 28.52		44.50 ± 32.18		77.00 ± 36.98		93.50 ± 53.07		100.50 ± 60.07	
Group III		0.00 ± 0.00		62.50 ± 37.73		95.00 ± 36.89		121.00 ± 43.38		130.00 ± 42.16	
p-value		< 0.05		> 0.05		> 0.05		> 0.05		> 0.05	
Significance		S		NS		NS		NS		NS	

NS – not significant, S – significant

*S. mutans* is a gram positive, facultative anaerobic bacteria commonly found in the human oral cavity and is a significant contributor to tooth decay.

In present study the daily use of chlorhexidine twice, for a week reduces the salivary *S. mutans* count highly significantly when comparing baseline with all sample levels which has been used for earlier studies.

Sekino S, Ramberg P, Uzel NG, Socransky S, Lindhe J (2003)^4^ in their study evaluated that daily use of chlorhexi-dine mouthrinse as an adjunct to careful mechanical tooth cleaning reduces the number of microorganisms that could be detected in saliva sample.

C Rindom Schiott, WW Briner, H Loe (1976)^2^ evaluated that the number of students with *S. mutans* present in saliva decreased significantly by treatment with chlorhexidine.

Magnetized water is the water treated with magnetic force, which itself contains magnetism. In other words, when water comprising the properties of minerals is kept in contact with a magnet, then magnetism passes into it and all the water gets magnetized.

Wevangti Vangra (2008)^5^―water ionization, when water is affected by electromagnetic vibrations, some molecules of water will separate to hydrogen ion (H+) and hydroxyl ion (OH^-^). Some hydroxyl ions will combine with minerals such as calcium and become calcium bicarbonate which has alkaline property. Magnetized water has pH value about antioxidant property in which, some hydroxyl ions (OH^-^) combine together and become H_2_O and oxygen ion (O). This oxygen ion can stop free radical cycle because it is negatively ion 7.6 to 8.5.

When some oxygen ion will combine together and become oxygen, this oxygen can dissolve immediately in that water. If we put the magnetized water in closed bottle, there are small bubbles that get attached to the walls of bottle. It is said ‘Water which has alkaline property, always has oxygen inside’. This gives energy to the cells, prevents development of anaerobic bacteria and stops their growth.

Since magnetized water is alkaline and also as *S. mutans* is anaerobic bacteria, therefore, its alkaline property stops the anaerobic bacteria to grow, thereby reducing the count.

When comparing the mean change of both groups, the fall in the *S. mutans* count was highly significant p < 0.001

The present study results demonstrate that group I shows more reduction in *S. mutans* count than group IIA.

Menendez A, Li F, Michalek SM, Childers NK (2005)^6^ in their study evaluated that lower concentration of chlorhexidine used in the US (0.12%) may not be sufficiently strong to reduce *S. mutans* (even in combination with hydrogen peroxide) compared with other concentration, i.e. 0.2%.

Also, the combination of chlorhexidine mouthrinse and hydrogen peroxide did not have a greater effect than chlorhexidine alone in decreasing the oral *S. mutans* or *Streptococci* levels.

When comparing the mean change between both the groups on Day 1 (morning and evening) in *S. mutans* count was highly significant. On day 4 (evening) and day 7, (morning) the fall in *S. mutans* count was significant p < 0.01 and p < 0.05 respectively and day 7 (evening) the count was not significant p > 0.05.

The results of the present study demonstrate that by day 7 evening the *S. mutans* count for group IIB was almost in par with group I.

The statistical comparison of mean change of *S. mutans* at various levels between group I and group III shows that on day 1 (morning and evening), day 4 (evening), day 7 (morning) in *S. mutans* count was highly significant statistically with p < 0.001.

The day 7 (evening) the fall in count was not significant with p > 0.05.

The present study results show that by day 7 (evening) the *S. mutans* count for group III comes almost in par with group I.

When comparing the mean change at various levels between group IIA and group IIB, the *S. mutans* count on day 1 (morning) was statistically nonsignificant p > 0.05.

On day 1 (evening) and day 4 (evening), the count was statistically significant with p < 0.05 and p < 0.01 respectively. On day 7 (morning and evening), the *S. mutans* count was also statistically significant with p < 0.05.

The present study shows that rinsing for 3 minutes with magnetized water has more reduction in salivary *S. mutans* count than rinsing for 1 minute.

The statistical comparisons of mean change of salivary *S. mutans* (in cfu) between all the samples of group IIB and group III show that on day 1 (morning) the result points toward the statistically significant difference with p < 0.05. On day 1 (evening), day 4 (evening), day 7 (morning) and day 7 (evening), the fall in *S. mutans* between both the groups was statistically nonsignificant (p > 0.05).

The study shows that no statistical difference was found in the *S. mutans* count when water was magnetized for 24 hours and 72 hours. Therefore, magnetizing water for 24 hours also seems to be satisfactory for reducing the *S. mutans* count but with variable results.

## SUMMARIES AND CONCLUSIONS

According to the present study:

- When comparing the antibacterial efficacy, Chlorhexi-dine has shown better reduction in *S. mutans* count than the magnetized water. Magnetized water has also shown reduction in *S. mutans* count and therefore, it can be used as an alternative to chlorhexidine
- The variables―dosage (10 ml) and frequency (twice daily)―are kept constant for all the groups and have significant effect on reducing the *S. mutans* count and plaque formation. Whereby, these parameters can be kept as standards for rinsing with magnetized water.

Chlorhexidine (0.2%) has shown more reduction in *S. mutans* count when rinsing for 1 minute than magnetized water.

When comparing between 24 hours magnetized water, more reduction in *S. mutans* count was seen in group rinsing for 3 minutes than 1 minute rinse.

When comparing between 24 hours magnetized water (3 minutes) and 72 hours magnetized water (3 minutes) equal reduction in *S. mutans* count was observed which was almost in par with 0.2% chlorhexidine.

- Taste of magnetized water was also well accepted by children.

As already proved, chlorhexidine is the ‘Gold Standard’ for antibacterial and antiplaque effects. Magnetized water has also shown good results for antibacterial effects and, therefore, can be used as an alternative measure to chlorhexidine.
